# Effects of Loading Conditions on Articular Cartilage in a Metal‐on‐Cartilage Pairing

**DOI:** 10.1002/jor.24426

**Published:** 2019-08-06

**Authors:** Christoph Stotter, Bojana Stojanović, Christoph Bauer, Manel Rodríguez Ripoll, Friedrich Franek, Thomas Klestil, Stefan Nehrer

**Affiliations:** ^1^ Faculty of Health and Medicine, Department for Health Sciences, Medicine and Research, Center for Regenerative Medicine Danube University Krems Dr. Karl‐Dorrek‐Str. 30 Krems A‐3500 Austria; ^2^ Department of Orthopedics and Traumatology LK Baden‐Mödling‐Hainburg Waltersdorfer Straße 75 A‐2500 Baden Austria; ^3^ AC2T Research GmbH Viktor Kaplan‐Straße 2 A‐2700 Wiener Neustadt Austria; ^4^ Faculty of Health and Medicine, Department for Health Sciences, Medicine and Research Danube University Krems Dr. Karl‐Dorrek‐Str. 30 Krems A‐3500 Austria

**Keywords:** cartilage, metal implants, tribology, wear, tribocorrosion

## Abstract

The aim of this in vitro study was to investigate the response of articular cartilage to frictional load when sliding against a metal implant, and identify potential mechanisms of damage to articular cartilage in a metal‐on‐cartilage pairing. Bovine osteochondral cylinders were reciprocally slid against metal cylinders (cobalt–chromium–molybdenum alloy) with several variations of load and sliding velocity using a microtribometer. The effects of different loads and velocities, and the resulting friction coefficients on articular cartilage, were evaluated by measuring histological and metabolic outcomes. Moreover, the biotribocorrosion of the metal was determined. Chondrocytes stimulated with high load and velocity showed increased metabolic activity and cartilage‐specific gene expression. In addition, higher load and velocity resulted in biotribocorrosion of the metal implant and damage to the surface of the articular cartilage, whereas low velocity and a high coefficient of friction increased the expression of catabolic genes. Articular cartilage showed particular responses to load and velocity when sliding against a metal implant. Moreover, metal implants showed tribocorrosion. Therefore, corrosion particles may play a role in the mechano‐biochemical wear of articular cartilage after implantation of a metal implant. These findings may be useful to surgeons performing resurfacing procedures and total knee arthroplasty. © 2019 The Authors. *Journal of Orthopaedic Research*® published by Wiley Periodicals, Inc. on behalf of Orthopaedic Research Society J Orthop Res 37:2531–2539, 2019

Orthopedic surgical procedures (e.g., partial surface replacement and total knee arthroplasty without patella resurfacing) create a metal‐on‐cartilage (MoC) interface in the affected joint. However, these procedures are associated with postoperative complications (e.g., progressive wear of the preserved articular cartilage).^1^, ^2^, ^3^ Part of the damage occurs through direct contact of the cartilage articulating against metal implants.^4^ However, the adjacent cartilage may be equally affected.^5^

Partial surface replacement using metal implants remains a salvage procedure in the treatment of focal cartilage defects or osteochondral lesions. After failure of primary treatment (e.g., bone marrow stimulation), focal resurfacing procedures can reduce pain and improve the range of motion.^6^, ^7^, ^8^ However, even with correct implantation, the remaining articular cartilage shows accelerated wear in some cases.^1^, ^9^

In total knee arthroplasty without patella resurfacing, the preserved cartilage on the patella articulates against the femoral component of the implant, creating a MoC bearing surface. This may lead to high contact pressures in the cartilage and progressive degenerative changes of the non‐resurfaced patella.^10^ Patients who do not undergo resurfacing of the patella at the index procedure exhibit significantly higher rates of reoperation versus those who undergo resurfacing, due to anterior knee pain and other patellofemoral complications.^11^, ^12^

Previous studies have investigated MoC interfaces.^4^, ^13^, ^14^ Compared with other orthopedic implant materials, cobalt–chromium (CoCr) tends to accelerate cartilage wear.^14^ In addition to accelerated cartilage wear, increased disruption of the extracellular matrix and decreased cell viability have been demonstrated in a MoC bearing system versus those reported in cartilage articulating against cartilage.^13^ Damage to the cartilage when articulating against a metal implant mainly occurred in the form of delamination between the superficial and middle zones.^4^

Recent studies focused on the mechanical effects of loading, whereas investigation of the biological responses is thus far limited. Cartilage wear has been linked to mechano‐biochemical mechanisms. Therefore, data on chondrocyte viability and gene expression are essential for an improved understanding of cartilage degeneration. Furthermore, biotribocorrosion, in the form of metal particles and ion release, may play a role in articular cartilage wear.

The primary objective of this study was to investigate the response of chondrocytes to mechanical load and the underlying mechanisms leading to the degeneration of articular cartilage in a MoC pairing. By applying different loads and sliding velocities, the study aimed at identifying critical loading parameters in a MoC tribological testing system. The secondary objective was to determine the biotribocorrosion of a cobalt–chromium–molybdenum (CoCrMo) alloy sliding against the articular cartilage.

Initially, it was hypothesized that higher load and sliding velocity under frictional loading against metal implants may lead to accelerated damage to the cartilage, along with altered gene expression levels and metabolic activity. Second, it was hypothesized that biotribocorrosion occurs in a MoC setup with frictional loading and may contribute to cartilage damage.

## MATERIAL AND METHODS

### Preparation of Materials

#### Preparation of CoCrMo cylinders

CoCrMo cylinders were obtained from cylindrical rods (6‐mm diameter). They were wet ground using silicon carbide grinding paper (500–4,000 mesh). Subsequently, the rod was polished using 3‐µm and 1‐µm paste, resulting in a final surface roughness of 15 nm (±2 nm). The length of the cylindrical samples was 10 mm.

#### Preparation of specimens

For the cartilage counterpart, cylindrical osteochondral plugs from bovine stifle joints were harvested under aseptic conditions. The animals were aged 18–24 months at the time of sacrifice, and joints were kept contained and cooled until dissection. The osteochondral plugs (8‐mm diameter, 15‐mm length) were harvested from the medial femoral condyle using a cutting tube (OATS; Arthrex Inc., Naples, FL) within 24 h after sacrifice. The anteroposterior orientation was marked to arrange the osteochondral cylinder, as previously described.^15^ Each knee yielded nine samples, which were washed with phosphate‐buffered saline (PBS) (Sigma‐Aldrich Chemie GmbH, Steinheim, Germany). Subsequently, to maintain viability, the samples were placed in Dulbecco's modified Eagle's medium containing 10% fetal bovine serum, supplemented with antibiotics (penicillin 200 U/ml; streptomycin 0.2 mg/ml) and amphotericin B 2.5 µg/ml (Sigma‐Aldrich Chemie GmbH) at 4°C until testing to maintain viability.

Bovine knees were allocated to three treatment groups with different loading variations using block randomization. In each group, three osteochondral plugs served as a baseline and were analyzed immediately after harvesting, while three other osteochondral plugs were treated with the prescribed loading variations. The remaining three osteochondral plugs were submerged in PBS for the duration of the tribological testing and served as free‐swelling controls, treated as a zero‐area condition. After testing, the osteochondral plugs were stored in PBS at 4°C until analysis within 24 h.

#### Tribometrological protocol

**Figure 1:**
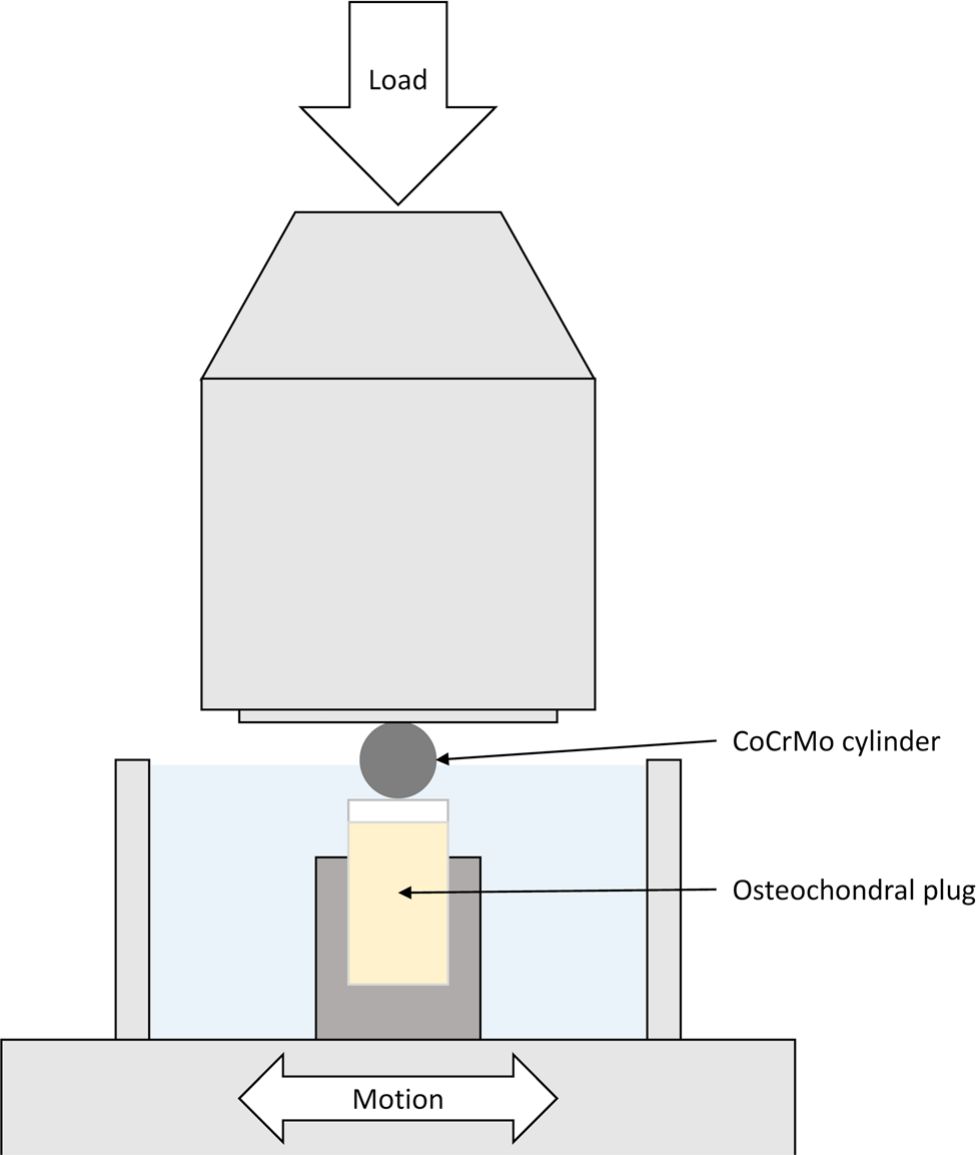
Schematic of the microtribometer (Tetra‐Falex MUST; Falex Tribology NV, Rotselaar, Belgium) and the tribometrological setup. The cobalt–chromium–molybdenum (CoCrMo) cylinders were mounted on the load cell, and the osteochondral cylinder was fixed in a custom‐made sample holder; the metal‐on‐cartilage interface was submerged in phosphate‐buffered saline (PBS, light blue). [Color figure can be viewed at wileyonlinelibrary.com].

All experiments were performed using a reciprocating microtribometer (Tetra‐Falex MUST; Falex Tribology NV, Rotselaar, Belgium) with a cylinder‐on‐plate and unconfined compression loading configuration (Fig. 1). Osteochondral cylinders were fixed in a custom‐made sample holder, and the CoCrMo cylinders were mounted onto the load cell. The system applied a prescribed normal load, which was maintained constant throughout the test. Reciprocal sliding was established by rubbing the CoCrMo cylinder against the articular cartilage immersed in the testing solution. The three groups with different loading variations to investigate the influence of sliding velocity and load were as follows: 0.1 mm/s, 1 N (group 1); 8 mm/s, 0.1 N (group 2); and 8 mm/s, 1 N (group 3). Fujifilm pressure measurements (Fujifilm Prescale; Fujifilm Corporation, Tokyo, Japan) were used to determine the size and shape of the contact area and contact pressure. The contact area showed an elliptical shape, owing to the convexity of the articular cartilage and the CoCrMo cylinders. Pressure measurements indicated contact pressures ≤2.5 MPa with a 1 N load. These values are comparable with femorotibial contact pressures observed during normal gait.^16^ Furthermore, a reciprocating relative movement of ±2 mm was applied to create a migrating contact, representing the situation in diarthrodial joints.^17^ The intermediate liquid used in all experiments was PBS (Sigma‐Aldrich Chemie GmbH). The tests were performed at room temperature, and the results were analyzed after 1 h.

### Analysis

#### Coefficient of friction (COF)

The COF was continuously monitored throughout the experiments. The time‐dependent COF was calculated from the ratio of the friction force to the normal force. The observed minimum value of the COF was denoted by μ_min_. The final steady state value, achieved after 1 h of testing, was denoted by μ_ss_. The average COF under steady state conditions was denoted by COF_av_.

#### Release of metal ions

After testing, the PBS was collected and analyzed for metal content without dilution using inductively coupled plasma (ICP) optical emission spectrometry (iCAP 7400 duo spectrometer; Thermo Fisher Scientific, Bremen, Germany). Calibrating solutions for CoCrMo were produced from ICP single‐element standards (Certipur; Merck KGaA, Darmstadt, Germany).

The limits of detection and quantitation were determined based on the measured intensity (Ib) and its standard deviation (SDb) from the blank sample. These limits were set to Ib + 3 SDb and Ib + 10 SDb, respectively.

#### Metabolic activity

The metabolic activity of chondrocytes within the cartilage of the osteochondral plugs was measured using an XTT assay kit (Cell Proliferation Kit II (XTT); Roche Diagnostics, Basel, Switzerland), as previously described.^15^ In short, cartilage was cut from the osteochondral grafts and bisected longitudinally, with equal distribution of the contact area on both pieces of articular cartilage. For metabolic activity, half of the cartilage was minced into small pieces and transferred onto a 24‐well plate, where the tissue weight for each sample was determined. Subsequently, the tissue was incubated in the XTT solution (1 ml medium, 490 µl XTT reagent, and 10 µl activation reagent) for 4 h at 37°C in 5% (v/v) CO_2_ in the air. After incubation, the XTT solution was removed and collected. Subsequently, 0.5 ml dimethyl sulfoxide was added to each sample, followed by incubation at room temperature for 1 h. The XTT and dimethyl sulfoxide solutions were pooled and transferred to a 96‐well plate. Absorbance and background wavelengths were measured at 492 and 690 nm, respectively, in sextuplicates using a multimode microplate reader (Synergy 2; BioTek Instruments, Winooski, VT) and Gen 5 software (BioTek Instruments). Absorbance was normalized to the wet weight of the tissue.

### Gene Expression

#### RNA isolation

The second half of the cartilage tissue was stored in RNAlater (Sigma‐Aldrich, St. Louis, MO) at 4°C for ≤7 days. For RNA isolation, the cartilage was minced into small pieces and transferred into tubes containing ceramic beads (MagNA Lyser Green Beads; Roche Diagnostics) with 300 µl lysis buffer (10 µl β‐mercaptoethanol + 290 µl RLT [Fibrous Tissue Kit; Qiagen, Hilden, Germany]). Homogenization of the cartilage tissue was achieved using the MagNA Lyser (6,500 rpm for 20 s ×4; Roche Diagnostics). RNA isolation was performed according to the instructions provided by the manufacturer. After elution in 30 µl RNAse‐free water, the RNA samples were stored at −80°C until complementary DNA (cDNA) synthesis.

#### Reverse transcription and real‐time quantitative polymerase chain reaction (RT‐qPCR)

Gene expression analysis was performed as previously described.^18^ Briefly, cDNA synthesis was performed using Transcriptor First Strand cDNA Synthesis Kit (Roche Diagnostics). RNA from bacteriophage MS2 was added to stabilize the isolated RNA during cDNA synthesis. RT‐qPCR was performed in triplicates using the LightCycler 96 (Roche Diagnostics). To determine the chondrocyte‐specific gene expression, we analyzed collagen type II (COL2A1) and aggrecan (ACAN). Notably, collagen type I (COL1A1), and matrix metalloproteinases 1 and 13 (MMP1 and MMP13) were used as markers of degradation. Target cycle threshold (*C*_t_) values were normalized to those obtained for the housekeeping gene glyceraldehyde‐3‐phosphate dehydrogenase and compared with the corresponding sample using the Δ*C*_t_ method with efficiency correction.

### Histology

For histological analysis, osteochondral plugs were submerged in 4% buffered formaldehyde solution (VWR, Radnor, PA) for ≤7 days, and decalcified for 4–6 weeks under constant agitation using the Osteosoft solution (Merck, Burlington, MA). After decalcification, the samples were embedded in Tissue‐Tek O.C.T. and stored at −80°C. Cryosectioning was performed using the CryoStar NX70 Cryostat (Thermo Fischer Scientific, Waltham, MA) to obtain 6‐µm sections. Subsequently, the samples were prepared for Safranin O staining and Fast‐green counterstaining. Histological images were captured using a Leica microscope DM‐1000 and processed using the Leica Manager software (Leica, Wetzlar, Germany).

### Statistical Analysis

All data are expressed as median and quartiles in box plots. Outliers and extreme outliers were defined as 1.5× and 3× the interquartile range outside the central box, respectively. The significance of differences observed between the treatment groups was analyzed using the nonparametric Mann–Whitney *U* test. Multiple comparisons were performed using the nonparametric Kruskal–Wallis test followed by Dunn's post hoc test in case of significance. The Kolmogorov–Smirnov test was used to confirm the normal distribution of Δ*C*_t_ values. Differences in gene expression were evaluated using one‐way analysis of variance with Bonferroni and Scheffé post hoc testing. All statistical analyses were performed using the GraphPad Prism software (Version 8.0.1; GraphPad Software Inc., San Diego, CA). A *p* < 0.05 denoted statistical significance.

## RESULTS

In total, each of the 12 bovine knees yielded nine osteochondral plugs, with a total number of 108 samples. Each group included four knees (*n* = 36).

### COF

**Figure 2:**
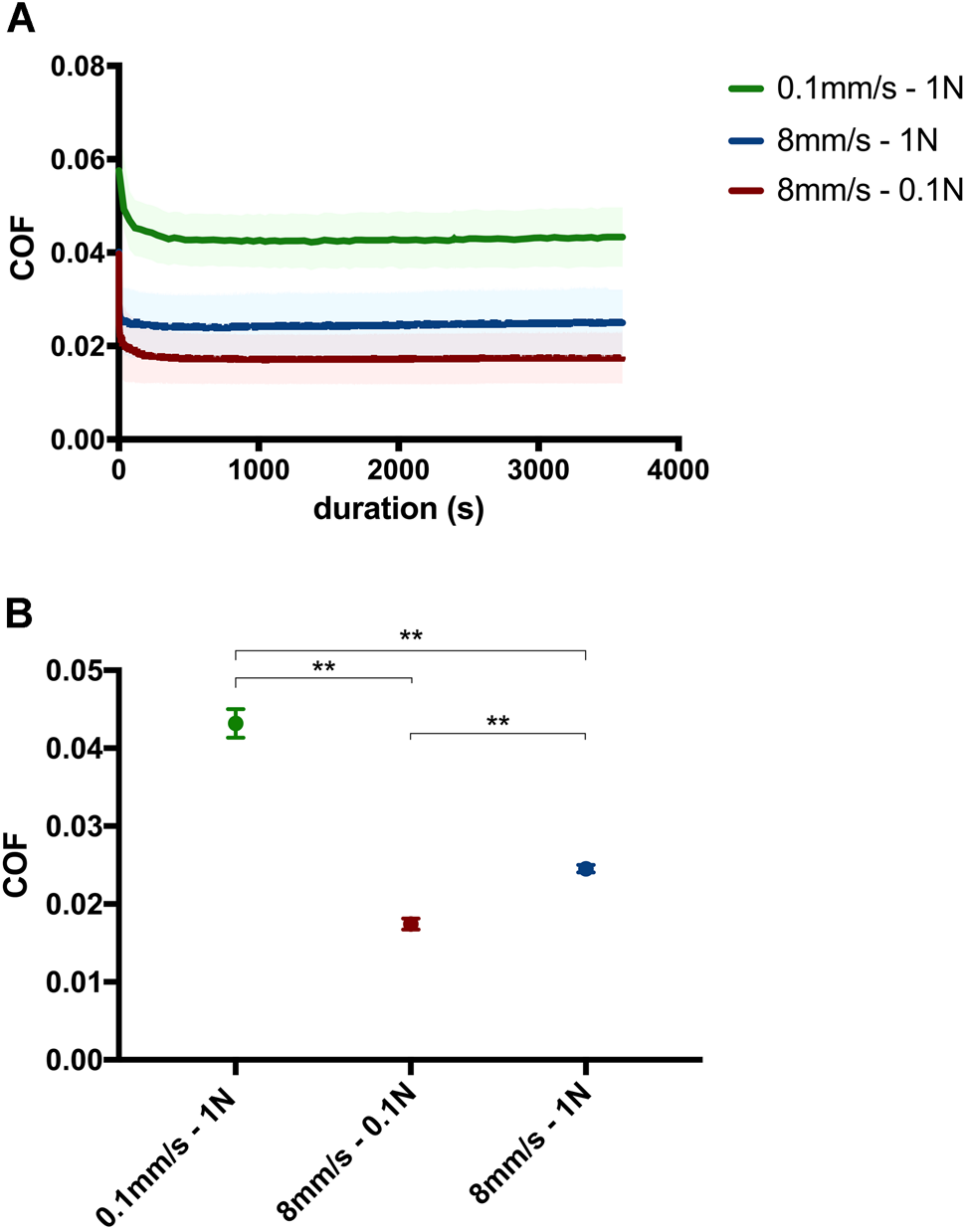
Coefficient of friction for all tested samples. (A) Time‐dependent coefficient of friction over the 1‐h testing period and (B) coefficient of friction (COF)_av_ for all groups along with statistical significance. **p < 0.0001. [Color figure can be viewed at wileyonlinelibrary.com].

In all experiments, the COF values stabilized after an initial decrease at the initiation of the reciprocating sliding motion (Fig. 2A). The range of COF_av_ was 0.017–0.043. High load and low velocities were associated with higher COF. Accordingly, group 1 (low velocity/high load) and group 2 (high velocity/low load) exhibited the highest (0.043 ± 0.006) and lowest (0.017 ± 0.005) COF, respectively. The COF of group 3 (high velocity/high load) was 0.025 ± 0.007. A statistically significant increase in the COF was observed between group 1 and the other groups (*p* < 0.0001), indicating an increasing COF with decreasing sliding velocity (Fig. 2B). Furthermore, the COF values in group 3 were significantly higher compared with those reported for group 2 (*p* < 0.0001), indicating an increasing COF with normal force.

### Release of metal ions

Metal ion release was only observed with high load and sliding velocity. Co ions (7.83 ± 3.63 ppb) were solely detected in group 3, indicating tribocorrosion of the CoCrMo alloy during mechanical testing. With lower sliding velocity and load, the levels of Co were below the detection threshold. Traces of Cr and Mo were detected in several samples; however, quantification was not possible. Therefore, the concentration ratios of each element in the solution could not be calculated.

### Metabolic activity

**Figure 3:**
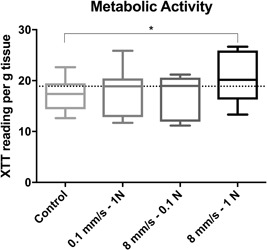
Metabolic activity (XTT reading per g tissue) of chondrocytes isolated from osteochondral cylinders after tribological testing with different loading variations and controls. Baseline expression levels are indicated by a horizontal dotted line. *p < 0.05.

Data regarding the metabolic activity of chondrocytes within the articular cartilage of the osteochondral plugs is presented in Figure 3. The baseline values (immediately after harvesting) were uniform throughout all knees and harvesting sites. The unloaded control samples showed decreased metabolic activity compared with the baseline values; however, this decrease was not statistically significant (*p* = 0.0566).

All tested groups showed increased metabolic activity compared with the unloaded controls, indicating mechanical stimulation. However, the difference was statistically significant only for group 3 (high velocity/high load) (*p* = 0.0052).

### Gene Expression

**Figure 4:**
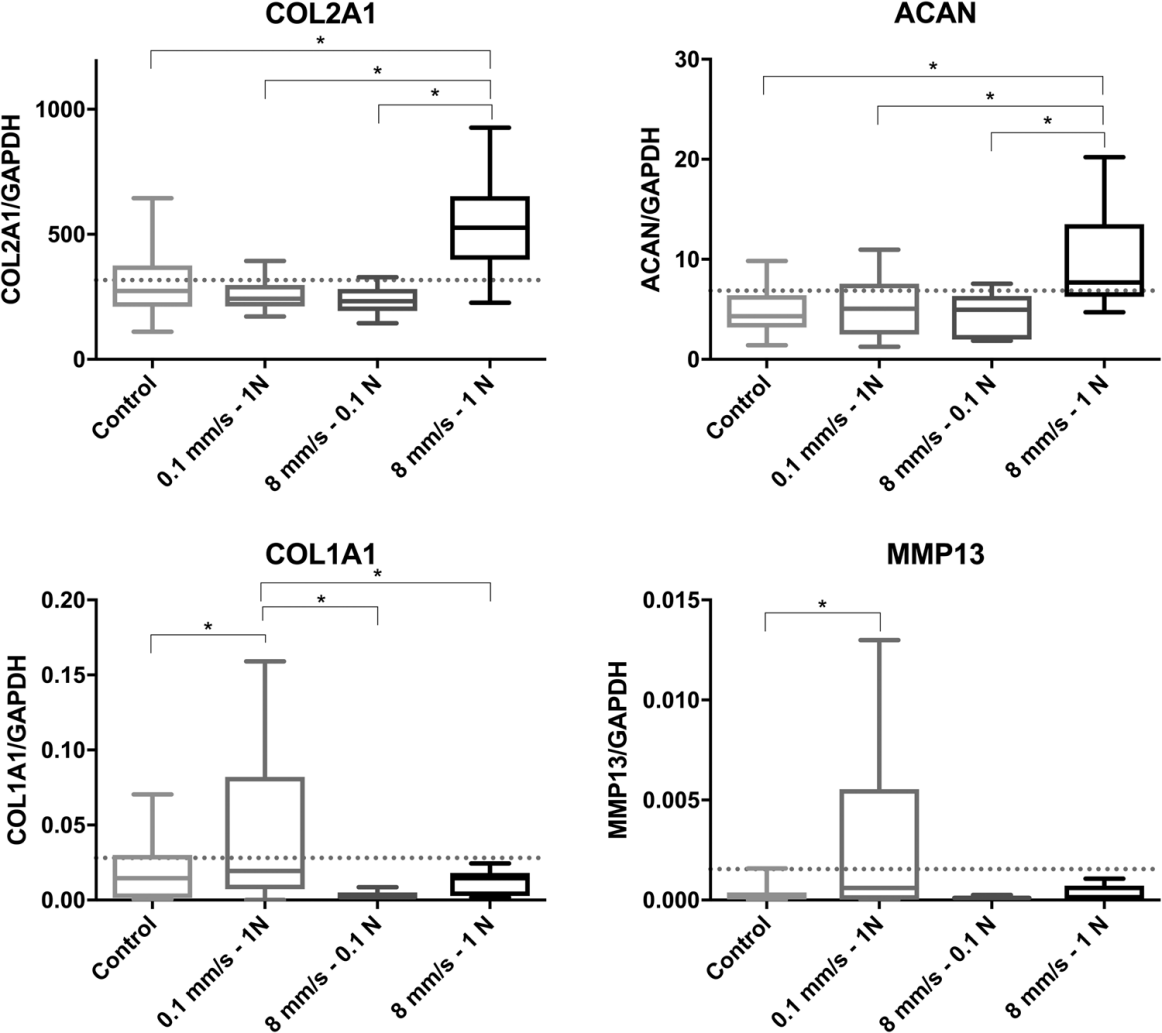
Gene expression of anabolic (COL2A1 and ACAN) and catabolic (COL1A1 and MMP13) cartilage‐specific genes in chondrocytes isolated from osteochondral cylinders after tribological testing with different loading variations and controls. The expression levels were normalized to the housekeeping gene glyceraldehyde 3‐phosphate dehydrogenase (GAPDH). Baseline expression levels are indicated by a horizontal dotted line. *p < 0.05.

The RT‐qPCR results demonstrated different gene expression profiles among chondrocytes exposed to different loading variations. The harvesting site did not alter the levels of messenger RNA (mRNA) expression. The gene expression of cartilage‐specific genes increased with high load and sliding velocity (i.e., 8 mm/s, 1 N). Particularly, the expression of COL2A1 and ACAN was significantly increased compared with all other loading variations and free‐swelling controls (Fig. 4). There was no difference detected between the other treatment groups and controls.

The mRNA expression levels of both catabolic genes (COL1A1 and MMP13) were significantly enhanced in group 1 (low velocity/high load) compared with those reported for groups 2 and 3 and unloaded controls. In all groups exposed to high velocity, the expression of COL1A1 and MMP13 remained low regardless of the load. The levels of MMP1 remained relatively stable without significant differences observed between the groups (data not shown).

### Histology

**Figure 5:**
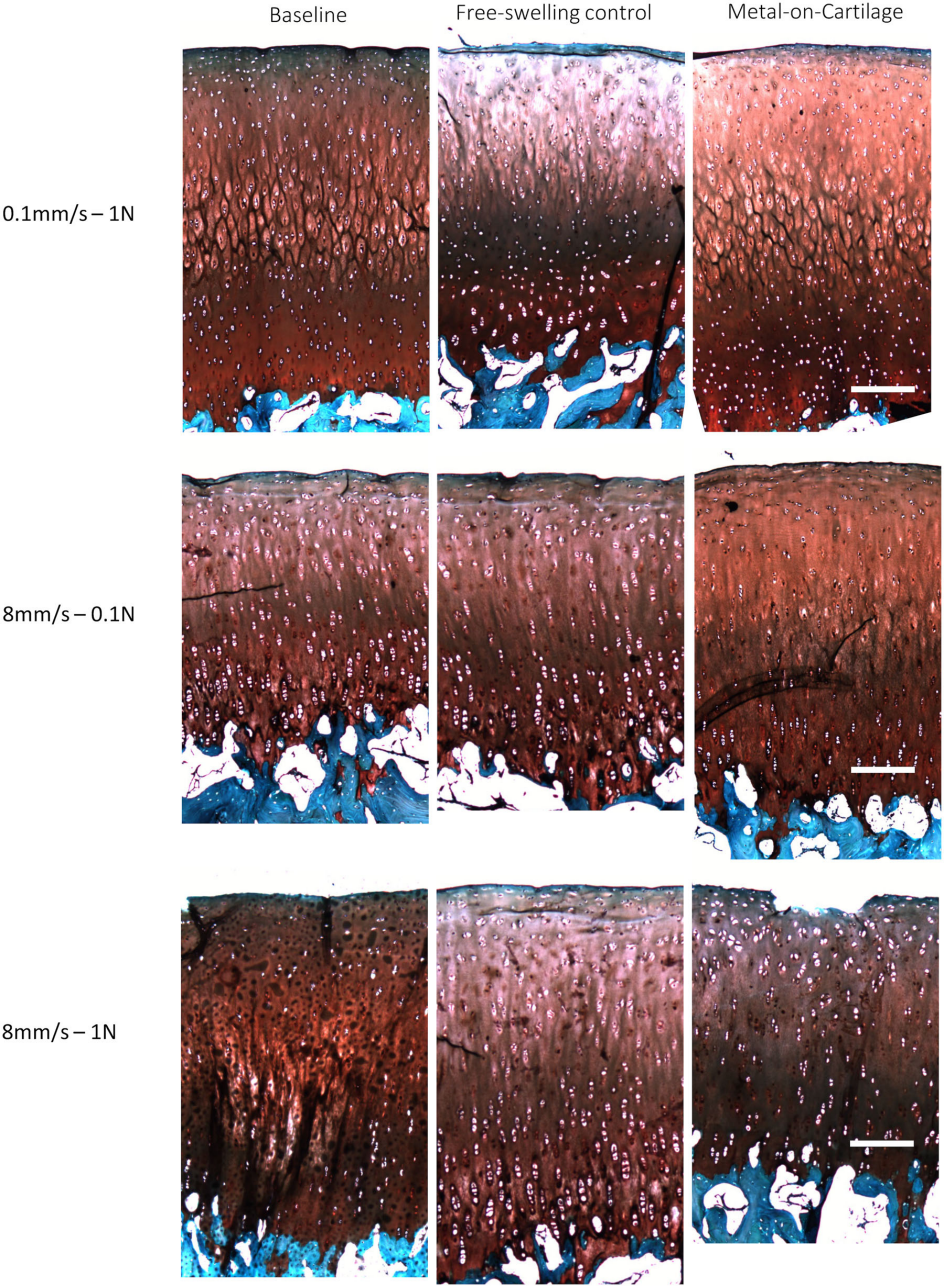
Representative histological cross sections of cartilage tissue with Safranin O and Fast‐green staining. Baseline samples, free‐swelling controls, and cartilage samples tested against metal implants are displayed for each treatment group; scale bar = 250 µm. [Color figure can be viewed at wileyonlinelibrary.com].

After testing against the metal counterpart, the integrity of the cartilage surface and its underlying matrix were analyzed using Safranin O and Fast‐green staining. Figure 5 shows cross sections of cartilage samples exposed to different loading variations and unloaded controls. In the baseline samples, the staining was intense and uniform throughout the tissue section. As expected, less staining was observed in the superficial layer due to the low level of proteoglycans.^19^

The control samples displayed less Safranin O staining in the middle zone compared with all load variations, indicating an extraction of substantial amounts of proteoglycans. Samples exposed to all loading variations did not show that extraction, implying mechanical stimulation in all groups. In group 3 (high velocity/high load), several samples showed damage to the surface and disruption of the superficial layer. However, such surface abrasions were not observed in the other treatment groups. This damage appeared delamination‐like between the superficial and middle zones, without deep ruptures or damage to the middle and deep zones (Fig. 5).

## DISCUSSION

### Different Responses of Articular Cartilage to Loading Variations in a MoC Tribological Pairing

The expression of cartilage‐specific genes (i.e., COL2A1, ACAN) was increased with higher load and velocity, indicating mechanical stimulation. Accordingly, samples exposed to those loading conditions showed higher metabolic activity. In our tribological analysis, we applied ≤1 N normal load and reciprocal sliding motion to mimic physiological dynamic compressive and shear loading. The measurements of contact pressure yielded consistent results with pressures occurring in human joints during activities of daily life (e.g., walking). Previously reported tibiofemoral contact pressures range from 1 MPa (during standing) to 10 MPa (during activities such as downhill running).^16^ In a finite element simulation, the maximum tibiofemoral contact pressure was 1.53 MPa in the healthy joint and increased slightly with a focal resurfacing implant.^20^

Mechanical stimulation of cartilage and chondrocytes has been described in in vitro, animal, and clinical studies.^19^, ^21^, ^22^, ^23^, ^24^ Generally, cyclic loading and joint motion are considered beneficial for producing the extracellular matrix and maintaining effective lubrication.

In our study, the expression of COL2A1 and ACAN was upregulated in response to 1 N load and 8 mm/s sliding velocity, suggesting an attempt to adapt the extracellular matrix to the mechanical conditions. A significant positive correlation between contact stress and COL2A1 has been described in a ball‐on‐plate configuration with a polyoxymethylene‐cartilage pairing.^21^ COL2A1 is the most abundant collagen found in articular cartilage. Therefore, the observed upregulation may indicate that the chondrocytes are adapting to a physiological stimulus. Schätti et al.^25^ also found a relationship between sliding velocity and gene expression in a ball‐on‐plate setup with a Teflon‐cartilage bearing. The present samples exposed to higher load and velocity also showed increased metabolic activity. Trevino et al.^13^ compared cartilage‐on‐cartilage and MoC and reported mechanical stimulation of cell activity in their cartilage‐on‐cartilage set‐up versus free‐swelling controls. For the MoC group, they reported significant loss in cell viability in the superficial zone, surface damage, and decreased Safranin O staining. However, they did not report on cell activity.

In our experiments, low velocity and higher COF also increased the expression of catabolic genes (COL1A1, MMP13). The increase of COF in response to low sliding velocity is established in the literature.^26^ However, the observed increase in the expression of these markers of cartilage degradation is a new finding. This increase occurs in response to very low velocity (0.1 mm/s). Thus, it is likely that such low velocity limits interstitial fluid support to a level equivalent to that of stationary contact. Such adverse loading conditions with the absence of adequate mechanical stimulation may occur during postoperative immobilization. Our findings illustrate the importance of postoperative management, continuous motion, and cyclic loading.

A migrating contact point has been shown to improve lubrication, allow rehydration of the cartilage, and improve cell viability at the contact point over time in cartilage explants.^17^ Cartilage deformation and contact stresses increased in response to increasing sliding velocity.^21^ In a study involving a stainless steel‐cartilage pairing, the COF was low with high sliding velocity (1–5 mm/s), regardless of the contact area. In contrast, low sliding velocity increased the COF.^27^ The COF remains low for longer when the area of contact is larger (e.g., congruent joints) and increases when the contact area decreases (e.g., after trauma or surgical procedures).^28^

Following the clinical observation of cartilage damage after surgical procedures (i.e., partial resurfacing, and total knee arthroplasty without patella resurfacing), we selected a MoC pairing.

Our histological analysis showed damage to the cartilage, appearing as surface disruption, in the same samples in group 3. However, the damage affected only the superficial zone and did not proceed into the middle and deep zones. The mechanism of damage appears to be delamination between the superficial and middle zones, with disruption of the top layer. The use of synovial fluid as lubricant provides boundary protection, facilitates interstitial fluid pressurization, and may prevent such damage.^17^

Oungoulian et al.^4^ observed cartilage damage in a MoC bearing system in the form of delamination of the top layer, rather than abrasive wear to the surface of the cartilage. They proposed a mechanism of failure due to subsurface fatigue between the superficial and middle zones, in which collagen fibers are randomly orientated. Furthermore, they demonstrated a higher rate of volumetric wear in one of their treatment groups (stainless steel) compared with glass.

Trevino et al.^13^ demonstrated accelerated cartilage wear (hydroxyproline content), disruption of the extracellular matrix, and decreased cell viability in the superficial zone in a MoC pairing compared with cartilage articulating against cartilage.

Notably, mechano‐biochemical mechanisms and biotribocorrosion may also contribute to cartilage damage. It is established that, in metal‐on‐metal bearing systems, corrosion and wear occur in the form of particles and metal ions.^29^ We demonstrated that CoCrMo alloys are prone to tribocorrosion when sliding against a much more compliant counterpart (i.e., articular cartilage).^30^ To the best of our knowledge, the impact of metal ion release of a CoCrMo alloy on articular cartilage damage during reciprocating sliding has not been described yet.

Our experiments confirmed biotribocorrosion in the form of Co ion release into the testing solution. ICP data showed release of Co exclusively in group 3 (8 mm/s; 1 N). Although Cr and Mo ions were detected in some samples, quantification was not possible. The higher concentrations of Co may be explained by the fact that Co forms dissolved ions, whereas Cr precipitates as solid oxide. Furthermore, the distribution of the components reflects the composition of the alloy (CoCrMo rod) (i.e., 65% Co, 27% Cr, and 5% Mo). In the majority of studies investigating CoCrMo alloys, Co represents >80% of the total metal ion release in corrosion and tribocorrosion tests.^31^, ^32^ Our findings may be relevant because chondrocytes are influenced by CoCrMo debris.^33^ In a cell culture model, CoCrMo particles were phagocytosed by chondrocytes and led to decreased cell viability through apoptosis. Exposure to wear debris induces the expression of pro‐inflammatory cytokines and other inflammatory mediators, which are involved in adverse local tissue reaction and periprosthetic osteolysis.^33^ Moreover, cytotoxic effects and altered gene expression levels for the components of CoCrMo have been reported in chondrocytes^34^ and various other cell types.^35^

The COF is affected by the concentration and viscosity of lubricants.^37^, ^38^ For instance, synovial fluid, was found to significantly reduce COF compared with Ringer's solution.^39^ The presence of synovial fluid in the contact interface of two cartilage surfaces may be beneficial, yielding lower COF compared with saline, water, and histidine buffer.^40^

However, unlike histidine buffer and saline, lubrication of the cartilage surface using synovial fluid was impaired at low temperatures.^40^ Therefore, in this study we used PBS.

The present results showed that different loading variations in a MoC pairing led to different COF values (0.017–0.043). In contrast, a cartilage‐on‐cartilage pairing is a quasi frictionless system. In the literature, the range of COF for normal joints and MoC pairings is 0.0005–0.04^37^, ^41^ and 0.005–0.57, respectively. This large range is attributed to the relative nature of the COF, which depends on numerous system properties (e.g., sliding velocity,^27^ normal load,^40^, ^41^ and viscosity^42^). Furthermore, the COF is influenced by the contact area,^28^ contact point (i.e., migrating vs. stationary^17^), contact stress,^43^ dwell time,^37^, ^40^ type of lubricant,^40^ and surface roughness.^4^

Caligaris et al.^17^ showed that the COF remains low with a migrating contact area and physiological loading for ≥1 h (the duration of our experiments). In agreement with previous findings,^27^ we observed an increase in the COF with decreasing sliding velocity. Interestingly, the expression of COL1A1 and MMP13 was increased in group 1, showing the highest COF (0.043). The observed increase in the expression of catabolic genes indicates the adverse effect of high friction. In a study investigating a cartilage‐on‐cartilage pairing, higher contact stress (0.5–3.15 MPa) resulted in higher COF and cartilage wear in native cartilage.^43^

Oungoulian et al.^4^ also reported the greatest damage with higher equilibrium COF. The COF in situ will not achieve the elevated equilibrium values observed under common testing conditions.^28^ In vitro tests showed a time‐dependent frictional response of articular cartilage, with the COF increasing over time under constant load.^26^ Frictional response correlated with interstitial fluid pressure. High interstitial pressure produced a low friction, whereas low pressure led to high friction.^44^ In vitro dynamic loading of articular cartilage for ≤1 h led a similar increase in COF to that observed with static loading.^26^ Therefore, dynamic loading alone cannot explain the markedly low COF observed in vivo. In principle, the application of higher load results in a significant decrease of COF in cartilage‐on‐cartilage and MoC set‐ups.^40^ In our study, we did not detect this effect, as the effect of sliding velocity was more pronounced.

### Limitations

There are several limitations in this study. First, we used PBS as testing solution, which is not a lubricant. Nevertheless, PBS remains the most commonly used fluid for tribological testing.^45^ Being aware that the introduction of synovial fluid can reduce the COF and alter gene expression,^39^, ^40^ we used PBS to minimize the influence of factors and generate more robust data.

Second, we used osteochondral plugs harvested from skeletally mature cattle. Considering the observed variability in the biological and mechanical properties of bovine and human tissues,^46^ caution should be exercised in extrapolating the present findings between species.

Lastly, all experiments were performed at room temperature. A higher temperature may alter the results. However, in a cartilage‐on‐cartilage pairing study, a change in temperature (24°C vs. 37°C) did not exert a significant effect on the COF under static and dynamic testing.^40^ Nevertheless, when using synovial fluid, a physiological temperature of 39°C for bovine samples should be applied.

In a MoC bearing system, certain loading conditions (8 mm/s, 1 N) stimulated the biosynthetic activity of chondrocytes. This was indicated by higher metabolic activity and expression of cartilage‐specific genes. Moreover, higher load and velocity caused biotribocorrosion of the CoCrMo alloy, which was confirmed through Co ion release. The metal ion release may play a role in the mechano‐biochemical wear of articular cartilage when sliding against a metal implant. Furthermore, low velocity and a higher COF increased the expression of catabolic genes. The findings of this study may contribute to the understanding of damage to articular cartilage when sliding against metal implants.
